# Loganic Acid, an Iridoid Glycoside Extracted from *Cornus mas* L. Fruits, Reduces of Carbonyl/Oxidative Stress Biomarkers in Plasma and Restores Antioxidant Balance in Leukocytes of Rats with Streptozotocin-Induced Diabetes Mellitus

**DOI:** 10.3390/life10120349

**Published:** 2020-12-15

**Authors:** Olha Dzydzan, Iryna Brodyak, Anna Sokół-Łętowska, Alicja Z. Kucharska, Natalia Sybirna

**Affiliations:** 1 Department of Biochemistry, Ivan Franko National University of Lviv, 4 Hrushevskyi St., 79005 Lviv, Ukraine; Olha.Dzydzan@lnu.edu.ua (O.D.); nataliya.sybirna@lnu.edu.ua (N.S.); 2 Department of Fruit, Vegetable and Plant Nutraceutical Technology, Faculty of Biotechnology and Food Science, Wrocław University of Environmental and Life Sciences, J. Chełmo’ nskiego 37/41, 51-630 Wrocław, Poland; anna.sokol-letowska@upwr.edu.pl (A.S.-Ł.); alicja.kucharska@upwr.edu.pl (A.Z.K.)

**Keywords:** loganic acid, diabetes mellitus, leukocytes, plasma, erythrocytes, carbonyl/oxidative stress biomarkers, antioxidant enzymes

## Abstract

The various complications related to diabetes are due to the alteration in plasma components and functional activity of blood cells, hence the search for preventive remedies that would ameliorate the clinical condition of patients is a relevant problem today. The main aim of the present study was to examine the antidiabetic potency and antioxidant effects of loganic acid (LA) in blood of diabetic rats. LA showed a restoration of balance between functioning of antioxidant defense system and oxidative stress in leukocytes without notable effects on blood glucose levels when administered orally to rats (20 mg/kg b.w./day) for 14 days. LA ameliorated antioxidant status in leukocytes, as indicated by increasing the content of reduced glutathione and activities of catalase, glutathione peroxidase and glutathione reductase along with decreasing levels of intracellular reactive oxygen species. In addition, we observed the ability of LA to protect against formation and accumulation of glycation and oxidation protein products and malondialdehyde derivates in plasma. Therefore, LA showed antioxidant properties that may have beneficial effects under diabetes. Such results may represent LA as one of the plant components in the development of new drugs that will correct metabolic and functional disorders in leukocytes under diabetes.

## 1. Introduction

Diabetes mellitus (DM) is one of the most common endocrine diseases that have been identified as one of the priority issues for national health systems around the world. The social significance of this problem is due to the fact that DM is associated with development of numerous concomitant diseases, early disability, and metabolic complications [1]. The various complications related to diabetes are determined by changes of blood components. Leukocytes are the major cells of the inflammatory and immune response that defends against different type of infection, that is why these cells are important object for investigation. Experimental studies have shown that peripheral blood cells and plasma components are exposed to the direct and indirect effects of high blood glucose concentrations in patients with diabetes [1,2]. Hyperglycemia, which occurs in type 1 DM, induces an increased formation of free radicals, especially superoxide anion radicals. These free radicals can interact with NO to produce peroxynitrite or other reactive oxygen species (ROS) [3]. Reactive radicals induce DNA damage and cause modification of proteins with subsequent formation of the advanced oxidation protein products (AOPPs) and introduction of additional carbonyl groups into proteins (also as oxidatively modified proteins (OMPs)), which are markers of oxidative stress. It was also shown that AOPPs can also trigger oxidative stress and further stimulate ROS generation [4]. In addition, ROS enhances the processes of lipid peroxidation (LPO) resulting in the formation of lipid peroxides and malondialdehydes. Thus, DM as a multifactorial pathology is associated with oxidative stress. Therefore, the alteration in plasma components and in morphofunctional state of blood cells can be explained by defects in the balance between levels of free radicals and antioxidants.

The other mechanism for the development of these complications is the formation of advanced glycation end products (AGEs). AGEs accumulate in long-lived proteins of tissues, causing crosslinking, developing inflammation and thickening of basement membranes [4].

Despite a large number of synthetic drugs already on the market, scientists continue to explore the mechanism of phytopreparations. Phytotherapy might provide significant support for established treatments at all stages of the disease. Medicinal plants that offer considerable pharmaceutical potential have been used for thousands of years and continue to provide new types of remedies [5].

In our previous research, we found antidiabetic and antioxidant effects of cornelian cherry (*Cornus mas* L.) extracts in rats with experimental diabetes [6]. Extracts of red and yellow fruits of cornelian cherry contain iridoids in significant amounts (73.5% and 88.2%, respectively) and one of its main iridoid glycosides is loganic acid (LA). Therefore, we decided to investigate whether LA is responsible for the manifestation of effects of the studied extracts.

Loganic acid was proven to be effective for treating rheumatoid arthritis [7], inflammation [8]. This iridoid glycoside possesses distinguished neuroprotective effects [9], defends against diet-induced hypertriglyceridemia, atherosclerosis [10] and has antiadipogenic effect [11]. The LA reduced total cholesterol in blood and lipid accumulation in the aortic wall of rats with streptozotocin-induced diabetes [12]. However, the antioxidant effects of LA in leukocytes of rats with DM have not been previously examined to date.

The aim of this study was to evaluate the impact of LA administration on glucose-related parameters (fasting blood glucose, response to glucose in the oral glucose tolerance test (OGTT) and glycated hemoglobin (HbA1*c*)). We also investigated blood parameters (number of erythrocytes and leukocytes, differential leukocyte count, hemoglobin content, mean cell hemoglobin (MHC), erythrocyte resistance to acid hemolysis), parameters associated with carbonyl/oxidative stress (levels of ROS, reduced glutathione (GSH), thiobarbituric acid reactive substances (TBA-RS), OMPs, AOPPs, AGEs) and activity of antioxidant enzymes (superoxide dismutase (SOD), catalase (CAT), glutathione peroxidase (GPx), glutathione reductase (GR)) in leukocytes of rats with streptozotocin-induced DM.

## 2. Materials and Methods

### 2.1. Chemicals

Streptozotocin (STZ), thiobarbituric acid (TBA), trichloroacetic acid, 2′,7′-dichlorodihydrofluorescein diacetate and 2,4-dinitrophenylhydrazine were obtained from Sigma-Aldrich (St. Louis, MO, USA). 5,5′-Dithiobis 2-nitrobenzoic acid (DTNB, Ellman’s reagent) was acquired from Acros Organics (Geel, Belgium). Acetonitrile for LC-MS was obtained from POCh (Gliwice, Poland). LA was purchased from Extrasynthese (Lyon Nord, France), and *trans*-caftaric acid from Cayman Chemical Company (Ann Arbor, MI, USA).

### 2.2. Plant Materials and Sample Preparation of Loganic Acid

Ripe yellow fruits of two cultivars of cornelian cherry (*Cornus mas* L.) cultivars, “Yantarnyi” and “Flava”, were collected from the Institute of the Arboretum in Bolestraszyce, near Przemyśl, Poland. The adequate voucher specimens (“Yantarnyi”—BDPA 14131; “Flava”—BDPA 8795) were deposited at the Herbariums of Arboretum in Bolestraszyce, Poland. Loganic acid extract was prepared at the Department of Fruit, Vegetable and Plant Nutraceutical Technology in Wrocław University of Environmental and Life Science (Wrocław, Poland), in according to Kucharska et al. [13].

### 2.3. Identification of Compounds by Liquid Chromatography-Mass Spectrometry (LC-MS)

Identification of compounds was achieved using the Acquity ultra-performance liquid chromatography (UPLC) system, paired with a quadrupole-time of flight (Q-TOF) MS instrument (UPLC/Synapt Q-TOF MS, Waters Corp., Milford, MA, USA), with an electrospray ionization (ESI) source [6]. The separation was performed on an Acquity BEH C18 column (100 mm × 2.1 mm i.d., 1.7 µm; Waters Corp., Milford, MA, USA). Iridoids and phenolic acids were explored in the negative mode before and after fragmentation.

### 2.4. Determination of Compounds by HPLC

HPLC-PDA method was performed using a Dionex system (Germering, Germany), equipped with an Ultimate 3000 diode array detector, EWPS-3000SI autosampler, LPG-3400A quaternary pump and a TCC-3000SD thermostated column holder. All compartments were controlled by Chromeleon v.6.8 software (Thermo Scientific Dionex, Sunnyvale, CA, USA) [6]. A Cadenza Imtakt column C5—C18 (75 × 4.6 mm, 5 µm) was used. Loganic acid and its isomers were detected at 245 nm, while phenolic acids were at 320 nm. Loganic acid and its isomers were expressed as LA, and isomers of caftaric acid as *trans*-caftaric acid. The results were expressed as mg/100 g dry weight (dw).

### 2.5. Animal Experiments

#### 2.5.1. Animal Study

The animal experiments were approved by the Ethics Committee of the Department of Biochemistry of the Faculty of Biology (Protocol N 10, 3 June 2019), Ivan Franko National University of Lviv, Ukraine. Experiments were performed in according with Directive 2010/63/EU of the European Parliament and the Council of 22 October 2010 on the protection of animals used for scientific purposes and the National Institutes of Health guide for the care and use of laboratory animals (NIH publications no. 8023, revised 1978). Experimental procedures and handling of the animals were carried out as stipulated by the guidelines for the humane use of animals.

#### 2.5.2. Induction of DM in Rats

Male Wistar strain rats weighing 130 ± 5 g were used in all experiments. Animals had free access to water and standard chow and were kept under a 12/12 h light/dark cycle. The room temperature and humidity were maintained automatically at about 22 ± 2 °C and 60 ± 5%, respectively. After one week of adaptation, rats were randomly divided into three groups containing eight animals each: group 1—control rats (healthy animals), group 2 (DM)—rats with STZ-induced DM, group 3 (DM + LA)—diabetic rats treated with LA. The animals of the control group were selected among intact animals, which had a glucose concentration in the range of 3.7–5.0 mmol/L.

DM was induced by a single intraperitoneal injection of rats with STZ (Sigma-Aldrich) at a dose of 60 mg/kg b.w. STZ was dissolved in freshly prepared 10 mM Na-citrate buffer (pH 4.5) and injected to animals fasted overnight. Blood samples were obtained from each animal from the tail vein 72 h post-injection and the blood glucose level was measured using the glucose oxidase method (Filisit diagnostics kit, Dnipro, Ukraine). STZ-treated rats with glucose levels over 12 mmol/L were considered as diabetic and used for further experiments.

#### 2.5.3. Experimental Design

On the 10th day after STZ treatment, rats with DM were randomly separated into 2 groups: DM (diabetic control) and DM + LA (diabetic experimental group + loganic acid). Once daily for 14 days, rats received the following substances by gavage: Control and DM groups—1 mL/rat of water, group DM + LA—1 mL/rat of aqueous solutions of LA at a dose of 20 mg/kg b.w. LA is represented by a mixture of three isomers of loganic acid extracted from yellow fruits of cornelian cherry. The dose and duration of administration were selected based on previous studies [6,10].

### 2.6. Measurements of Body Weight and Weight Gain

The body weight (BW) in each group was estimated on days 0 (initial) and 14 (final) of the experiment. The growth rate of body weight (gain, %) was calculated using the formula Gain (%) = {(BW on the 14 day (g) − BW on day 0 (g))/BW on day 0 (g)} × 100.

### 2.7. Fasting Blood Glucose and Oral Glucose Tolerance Test (OGTT)

The levels of fasting blood glucose were measured at days 0 (initial) and 14 (final) of administration of LA using the glucose oxidase method (Filisit diagnostics kit). The blood samples were collected from the tail vein of each animal, which were fasted overnight and have free access to water. The OGTT was performed at the end of experiment after 16 h of fasting. Glucose solution was administered to experimental rats at a dose 1 g/kg b.w. Blood samples were taken before glucose loading (baseline) and after 30, 60, 90 and 120 min. As the criterion shows a general increase in glucose concentrations after glucose consumption, the index of the area under the glycemic curve (AUC*glu*) was calculated [14].

### 2.8. Blood Collection

The duration of extracts administration was 14 days. At the end of experimental period, rats of all groups were anesthetized using deep ether anesthesia and euthanized by decapitation. Blood was collected into vials with heparin. Then, 2 mL of blood was centrifuged at 3000 rpm to obtain plasma. Plasma was frozen and stored at −20 °C for further measurements. Red blood cells (RBCs) were washed three times with cold (4 °C) phosphate buffered saline (PBS, 137 mM NaCl, 2.7 mM KCl, 10 mM Na_2_HPO_4_ × 7H_2_O, 1.8 mM KH_2_PO_4_, pH 7.4).

### 2.9. Isolation of Blood Leukocytes

Leukocytes were isolated from blood by centrifugation over a gradient of ficoll-triombrast density (*r* = 1.076–1.078). Then, the cells were washed three times with cold (4 °C) PBS.

Leukocytes (2.5 × 10^6^) were lysed in 150 μL of the buffer composed of 0.5% Triton X-100, 100 mM KCl, 5 mM MgCl_2_, 2 mM EGTA, 25 mM Tris, pH 7.5 and a protease inhibitor cocktail (Sigma). The lysates of leukocytes were centrifuged at 10,000 rpm for 10 min at 4 °C to remove cell debris. Supernatants were collected and used for biochemical assays. The protein content in lysates of leukocytes and plasma was determined according to Lowry’s method [15].

### 2.10. Blood Parameters

#### 2.10.1. Parameters Related to Erythrocytes

RBCs count was carried out manually using a counting chamber. Hemoglobin concentrations in whole blood were measured using the cyanmethemoglobin method and mean cell hemoglobin (MCH) was determined according to Souza et al. [16]. Evaluation of level of glycated hemoglobin (HbA1*c*) was based upon the generation of 5-hydroxymethylfurfural (5-HMF) from ketoamine after treatment with oxalic acid and its subsequent reaction with thiobarbituric acid (TBA) to form a coloured adduct. The method for analysis of RBCs resistance to acid hemolysis was described earlier [17]. The erythrocyte hemolysis peak, hemolysis duration and ratio of mixed-age populations of erythrocytes (percentage) were evaluated.

#### 2.10.2. Leukocyte Related Parameters

The total number of white blood cells was calculated manually using a counting chamber. The differential leukocyte count was performed in painted smears of peripheral blood by the Romanovsky-Giemsa method [18]. About 200 cells were analyzed per sample to quantify the percentage rate of individual forms of cells.

### 2.11. Carbonyl/Oxidative Stress Markers in Plasma and Leukocytes

#### 2.11.1. Quantification of Reactive Oxygen Species (ROS) in Leukocytes

Leukocytes (2.0 × 10^5^) were incubated with 5 µΜ 2′,7′-dichlorodihydrofluorescein diacetate (H_2_DCFDA) at 37 °C for 30 min in the dark. After incubation leukocytes were washed twice with PBS and immediately analyzed using a fluorescence microscope (Nikon Optiphot 2, Tokyo, Japan). Fluorescence microscopic images were processed using Image J. The ROS production was calculated by the dividing total image 2′,7′-dichlorofluorescein (DCF) fluorescent intensity by the total number of leukocytes and was expressed in DCF fluorescence/cell (a.u.—arbitrary unit).

#### 2.11.2. Reduced Glutathione (GSH)

GSH was measured following the method described by Rizvi et al. [19], based on the property of GSH to reduce DTNB, forming a yellow-colored anionic product with absorbance maximum at λ = 412 nm. A molar extinction coefficient for nitrobenzoate ion was 13,600. The concentration of GSH was expressed in nmol/mg of protein.

#### 2.11.3. Lipid Peroxidation (LPO)

The degradation product of LPO, malondialdehyde (MDA), reacts with TBA at 100° C to form colored complex. The level of MDA was expressed as nmol/mL of plasma or as pmol/million of leukocytes using 156,000 M^−1^ cm^−1^ as a molar absorption coefficient at λ = 532 nm [20,21].

#### 2.11.4. Assay of Oxidatively Modified Proteins (OMPs)

The level of OMPs was determined following the method described by Demkovych et al. [22] with a slight modification [20]. The method is based on spectrophotometric detection of hydrazones formed in the reaction of aldehyde and ketone groups of aliphatic amino acid residues with the 2,4-dinitrophenylhydrazine that absorb at λ = 370 nm (OMP_370 nm_—OMPs of neutral characteristics) and at λ = 430 nm (OMP_430 nm_—OMPs of basic characteristics). Both OMP_370_ and OMP_430_ were expressed as nmol/mg of protein with molar absorption coefficient of 22,000 M^−1^ cm^−1^ and 16,800 M^−1^ cm^−1^, respectively [23].

#### 2.11.5. Assay of Advanced Oxidation Protein Products (AOPPs)

Determination of AOPPs, generated through the reaction of proteins with chlorinated oxidants such as chloramines, was performed following to Witko-Sarsat et al. [24] and Kalousova et al. [25] using spectrophotometric detection. The absorbance of the reaction mixture was read at λ = 340 nm against the blank. The chloramine-T standard solutions (0 to 100 µmol/L) used for calibration and concentration of AOPPs was expressed in chloramine-T units (nmol/mg of protein).

#### 2.11.6. Assay of Advanced Glycation End-Products (AGEs)

The level of AGEs in blood plasma and in leukocytes was determined according to the method described by Putta and Kilari [26]. Plasma was diluted with PBS (pH 7.4) in a ratio 1:50. The level of AGEs in plasma and in leukocytes was determined by measuring the fluorescence at an excitation wavelength of λ = 370 nm and an emission wavelength of λ = 440 nm. Bovine serum albumin solution (1 mg/mL) was used as a standard and its fluorescence intensity was defined as one unit of fluorescence. The fluorescence intensity was expressed in arbitrary units (AU) per mg of protein.

### 2.12. Antioxidant Enzymes Assays

The activity of SOD was determined according to the method described by Kakkar et al. [27]. One unit of SOD activity was defined as the amount of enzyme causing 50% inhibition of the reduction to formazan observed in the blank. The results were expressed as U/mg of protein.

The activity of CAT was estimated by decreasing the color intensity of the complex forming H_2_O_2_ with molybdenum salt [28]. Measurements were carried out at 410 nm. One unit of CAT activity was expressed as nmol of H_2_O_2_/min × mg of protein.

The activity of GPx was determined by the rate of oxidation of GSH before and after incubation of the sample with tertiary butyric hydroperoxide. The reduction of GSH was observed in the color reaction with DTNB [29]. GPx activity was expressed in μmol/min × mg of protein.

The activity of GR was measured by decreasing in absorbance due to the oxidation of NADPH, which is required for the reduction of oxidized glutathione at 340 nm [30]. One unit of GR activity was calculated as one nmole of NADPH/min × mg of protein using a molar absorption coefficient of 6.22 × 10^3^ M^−1^ cm^−1^.

### 2.13. Statistical Analysis

The results were calculated using Excel 2013 (Microsoft, Redmond, WA, USA) and expressed as mean ± standard error of the mean (M ± SEM). Analysis of variance (ANOVA) followed by Tukey’s post hoc multiple comparison test was used for data analysis and was performed in GraphPad Prism 8.0 (GraphPad Inc., San Diego, CA, USA). Differences between the groups were considered statistically significant at *p* < 0.05.

## 3. Results

### 3.1. Qualitative and Quantitative Analysis of Compounds of the Purified Loganic Acid Extract

The purified LA extract was prepared from yellow cornelian cherry fruits as described in the Materials and Methods section. The results of the identification and the content of the compounds (mg/100 g dw) of this extract are presented in Table 1.

In total, we determined one main compound with three isomers from the iridoids group and additionally one compound with two isomers from the phenolic acid group. Among the iridoids, there were three isomers of LA identified by their UV spectrum peak at 246 nm. These isomers had pseudomolecular ions at *m/z* 375 [M–H]^−^ and fragment ions at *m/z* 213 [M–H–162]^−^. We previously described the identification results for three identified isomers of LA in Cornelian cherry fruits [6]. The second compound in the tested extract was caftaric acid (*O*-caffeoyltartaric acid) with two isomers with UV spectra peaks at 327 and 328 nm. These compounds gave a [M–H]^−^ ion at *m/z* 311 and the most prominent [M–H–132]^−^ ion at *m/z* 179 after loss of a tartaric acid moiety (132 Da), as well as fragment ions at *m/z* 149 (tartaric acid) and at *m/z* 135 derived from the decarboxylation of caffeic acid. The two caftaric acid isomers were identified in Cornelian cherry fruit for the first time.

The amount of LA isomers was 97.6% of total bioactive compounds in purified extract (Table 1). The content of LA isomers was about five and seven times more than the content of these isomers in Cornelian cherry extracts from yellow and red fruits, respectively [6]. In addition, the amount of LA isomers in these extracts was 300 times more than in the unpurified extracts from fresh Cornelian cherry fruits [13]. Due to the fact that the studied extract has a high concentration of LA and its isomers (up to 74 g/100 g) and low concentration of caftaric acid (only 1.8 g/100 g), the extract was administered to animals with DM to estimate the effectiveness of LA.

### 3.2. Loganic Acid and Antidiabetic Effects

**Figure 1:**
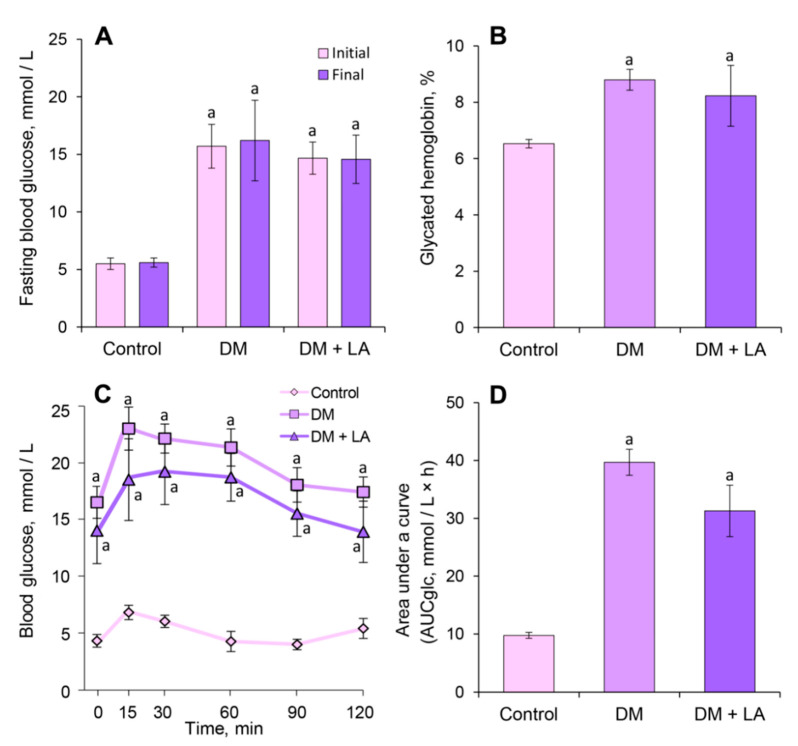
Effect of LA on glucose related parameters of diabetic rats after 14 days of administration: (**A**) fasting blood glucose, mmol/L; (**B**) glycated hemoglobin, % of total hemoglobin; (**C**) oral glucose tolerance test, mmol/L blood glucose levels and (**D**) the area under the curve after glucose load of rats, mmol/L × h. The results are shown as the mean ± SEM (*n* = 8). Designations: ^a^—*p* < 0.05 compared to the control group.

LA treatment of diabetic animals did not show effects on fasting blood glucose levels (Figure 1A). According to the obtained data, diabetic control rats showed a 35% increase in the HbA1*c* level compared to control rats, while the administration of LA to animals with DM showed no significant decrease in the level of HbA1*c* (Figure 1B).

The blood glucose levels of the LA-treated group were not different compared to the diabetic rats (Figure 1C). When the glycemic response of all the groups was expressed as the AUCglc (Figure 1D), the values of AUCglc were significantly high in the diabetic group and in the LA-treated diabetic rats 4.1- and 3.2-fold, respectively, versus controls. Thus, the administration of LA showed no hypoglycemic effect in diabetic rats.

### 3.3. Loganic Acid Alter Body Weight and Erythrocyte-Related Parameters

**Figure 2:**
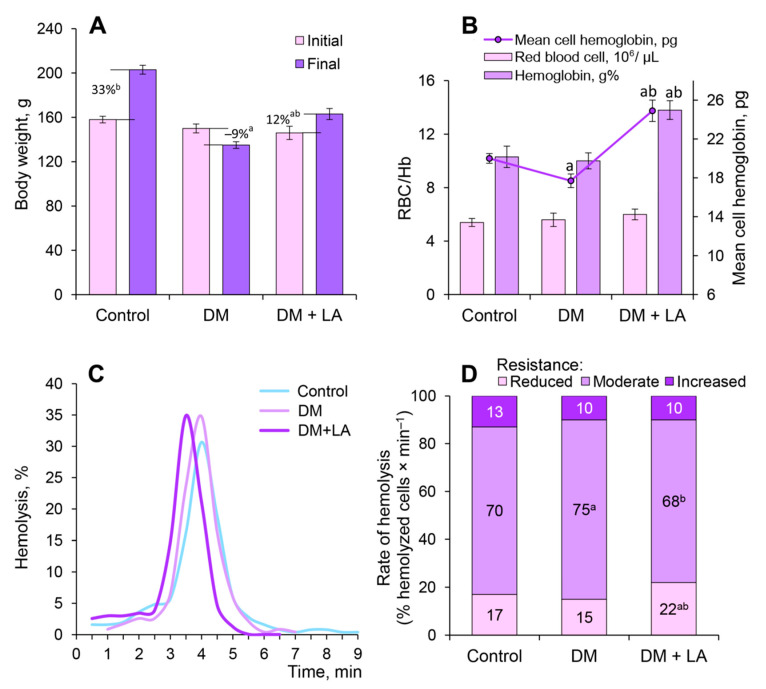
Effect of LA treatments on weight gain and erythrocyte related parameters of the diabetic rats: (**A**) the body weight gain, %; (**B**) number of red blood cells, 10^6^/μL, hemoglobin content, g% and mean cell hemoglobin, pg; (**C**) typical erythrograms, and (**D**) the ratio of mixed-age populations of erythrocytes of diabetic rats after 14 days of administration of LA. The results are shown as the mean ± SEM (*n* = 8). Designations: ^a^—*p* < 0.05 compared to the control group; ^b^—*p* < 0.05 compared to the DM group.

During the 14 days of the experimental period, diabetic rats showed a significant decrease in body weight compared to control animals; however, oral administration of LA led to a 12% increase in body weight in animals with diabetes (Figure 2A).

The total number of RBCs did not undergo significant changes in LA-treated diabetic rats, but the hemoglobin concentration and MCH were significantly increased by 38% and 40%, respectively, in comparison with the diabetic rats (Figure 2B). It is known that the MCH depends upon the volume of RBCs and their saturation degree with hemoglobin. Therefore, our data suggests that LA has normochromic effects in animals with DM.

The distribution of erythrocytes according to their resistance to the acid hemolytic agent was investigated in all experimental groups. In the control group of animals, the erythrogram is characterized by a rapid increase in hemolysis of the curve after the 3rd min with a maximum on the 4^th^ min and a gradual slow decrease to zero from 7th to 9th min (Figure 2C). When the erythrogram was expressed as the ratio of mixed-age populations of erythrocytes (Figure 2D), the rate of hemolysis of reticulocytes and erythrocytes with reduced resistance aged over 40 days was 17% (the segment of erythrogram from 1.5 to 3.0 min). The functional mature RBCs with moderate resistance aged 20–40 days was 70% (the segment of erythrogram from 3.5 to 4.5 min), and young erythrocytes with increased resistance aged up to 20 days was 13% (the segment of erythrogram from 5.0 to 7.5 min). We found reduced resistance of functional mature RBCs to the action of the acid hemolytic agent under conditions of DM (Figure 2C,D). After the administration of LA, the hemolysis peak shifted to the left and the hemolysis duration was decreased (Figure 2C). The mixed-age populations of erythrocytes of the LA-treated diabetic group showed an increase in the rate of hemolysis of reticulocytes and RBCs with reduced resistance by about 1.5-fold and significantly decrease the rate of hemolysis of functional mature RBCs with moderate resistance by 12%, as compared to the diabetic group (Figure 2D).

With the administration of LA, there was no observed significant increase in the total number of leukocytes as compared to the STZ-treated group, and the differential leukocyte count also did not differ from those in the control and diabetic groups (Table 2), indicating that the extract was not toxic to white blood cells.

### 3.4. Loganic Acid Changes GSH Content and OMPs in Plasma

**Figure 3:**
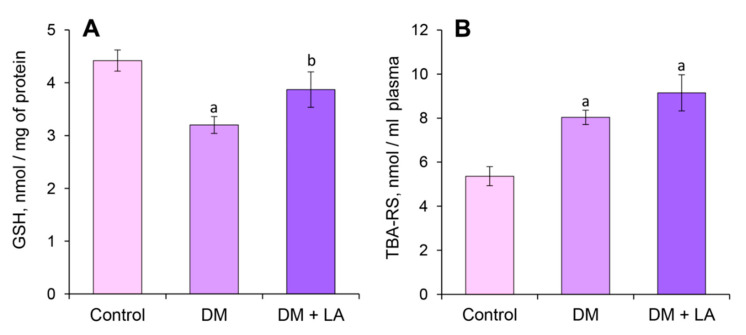
Effects of LA treatments (dose of 20 mg/kg b.w.) on concentrations of reduced glutathione (GSH), nmol/mg (**A**) and TBA-reactive substances (TBA-RS), nmol/mL (**B**) in plasma under DM. The results are shown as the mean ± SEM (*n* = 8). Designations: ^a^—*p* < 0.05 compared to the control group; ^b^—*p* < 0.05 compared to the DM group.

The plasma GSH level decreased significantly by 28% in rats with DM. Administration of LA caused a 21% increase in GSH levels compared to the nontreated diabetic group (Figure 3A).

The plasma TBA-RS level did not undergo significant changes in the LA-treated diabetic rats compared to the animals with DM (Figure 3B). This biomarker of LPO intensity was increased significantly by 50% and 70% in the diabetic rats and the LA-treated diabetic rats, respectively, compared to the control rats (Figure 3B).

**Figure 4:**
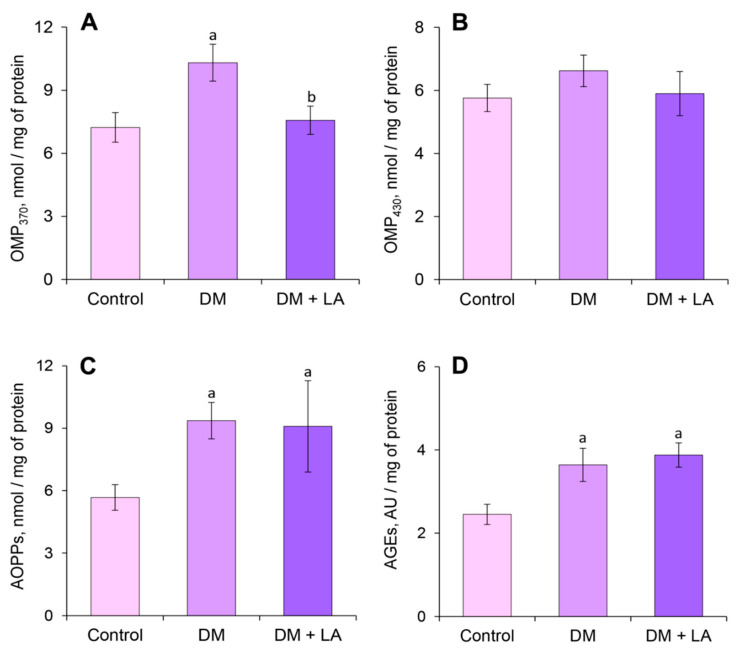
Effects of LA treatments on plasma oxidative stress-related parameters of diabetic rats: oxidative modifications of proteins (OMP) of neutral (**A**) and basic (**B**) character, nmol/mg; (**C**) advanced oxidation protein products (AOPPs), nmol/mg and (**D**) advanced glycation end products (AGEs), AU/mg in rats’ blood plasma under DM after 14 days of administration of LA. The results are shown as the mean ± SEM (*n* = 8). Designations: ^a^—*p* < 0.05 compared to the control group; ^b^—*p* < 0.05 compared to the DM group.

The level of OMPs of neutral characteristics increased by 42% in blood plasma under diabetic conditions compared to the control group (Figure 4A). Administration of LA to animals with DM decreased the levels of OMPs of neutral character by 27% compared to the nontreated diabetic group (Figure 4A). The level of OMPs of basic nature did not undergo significant changes in the diabetic rats and in the LA-treated diabetic rats (Figure 4B). The plasma level of AOPPs and AGEs significantly increased in the diabetic group by 65% and 49%, respectively (Figure 4C,D). After treatment with LA, the AOPPs and AGE levels did not differ from the nontreated diabetic rats (Figure 4C,D).

### 3.5. Loganic Acid Enhances an Antioxidant Defense System of Leukocytes

**Figure 5:**
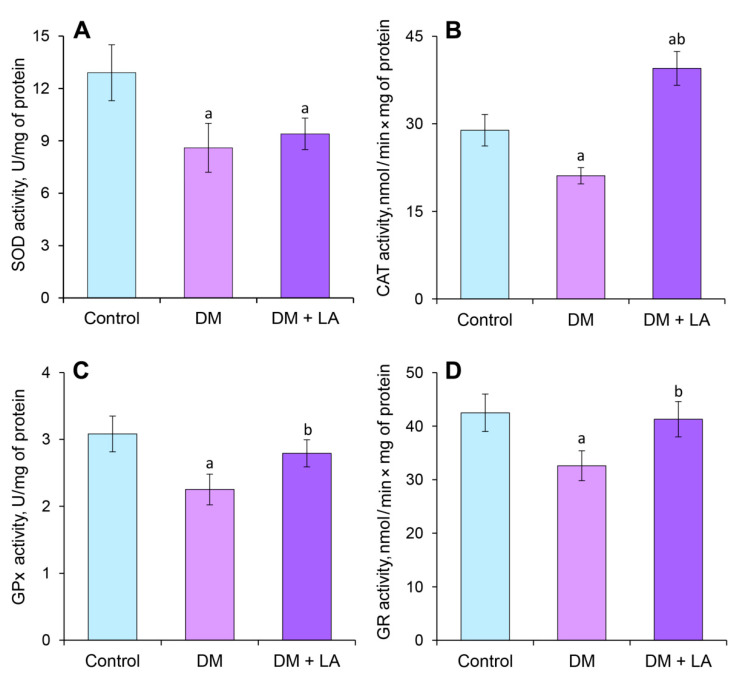
Effects of LA on activity of antioxidant enzymes in leukocytes: (**A**) SOD, superoxide dismutase; (**B**) CAT, catalase; GPx, (**C**) glutathione peroxidase and (**D**) GR, glutathione reductase. The results are shown as the mean ± SEM. Designations: ^a^—*p* < 0.05 compared to the control group; ^b^—*p* < 0.05 compared to the DM group.

Diabetes is accompanied by changes in the antioxidant defense system. Altered antioxidant enzyme activities were shown in leukocytes of a group of diabetic animals. A significant decrease was observed in the activity of SOD and CAT on 34% and 27%, respectively, compared to the control group (Figure 5A,B). Such changes were also observed in the activity of glutathione-dependent enzymes: the activity of GPx reduced by 27% and GR by 23% compared to the control group (Figure 5C,D).

The administration of LA to animals with DM caused an 85% increase in the activity of CAT (Figure 5B) but no significant change was shown in the activity of SOD compared to the non-treated group (Figure 5A). At the same time, leukocytes from diabetic animals treated with LA showed an increase of GPx and GR activity by 24% and 27%, respectively (Figure 5C,D).

### 3.6. Loganic Acid Alleviates Content of Carbonyl-Oxidative Stress Metabolites in Leukocytes

**Figure 6:**
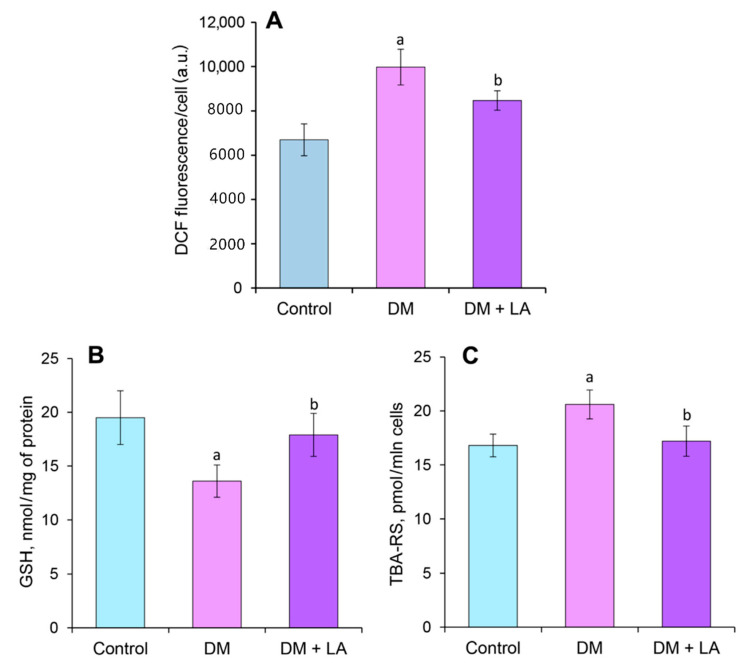
Effects of loganic acid (LA) (daily dose of 20 mg/kg b.w. for 14 days) on intracellular ROS level, DCF fluorescence/cell, (a.u.) (**A**); concentrations of reduced glutathione (GSH), nmol/mg (**B**) and TBA-reactive substances (TBA-RS) of lipid peroxidation, pmol/mln cells (**C**) in leukocytes of rats. The results are shown as the mean ± SEM (*n* = 8). Designations: ^a^—*p* < 0.05 compared to the control group; ^b^—*p* < 0.05 compared to the DM group.

**Figure 7:**
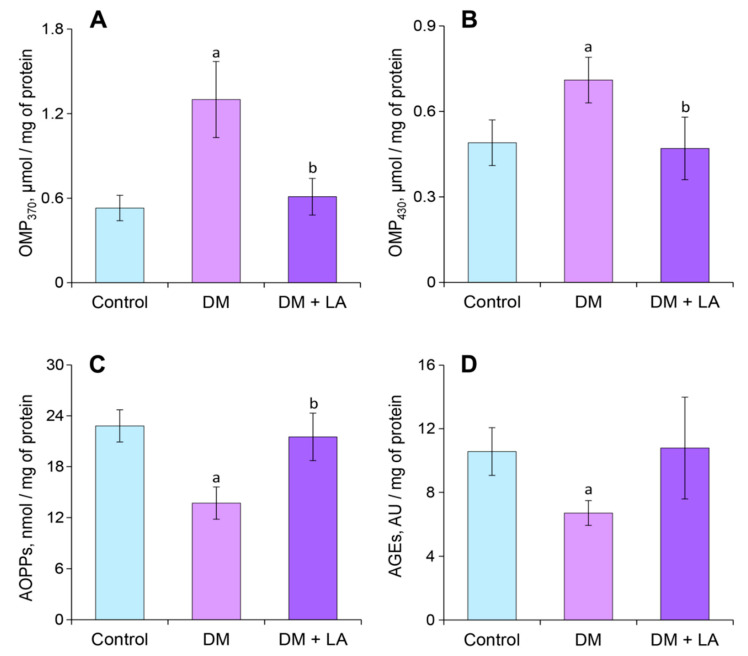
Effects of LA treatments on oxidative stress-related parameters in leukocytes of diabetic rats: oxidative modifications of proteins (OMP) of (**A**) neutral and (**B**) basic character, μmol/mg; (**C**) advanced oxidation protein products (AOPPs), nmol/mg and (**D**) advanced glycation end products (AGEs), AU/mg. The results are shown as the mean ± SEM (*n* = 8). Designations: ^a^—*p* < 0.05 compared to the control group; ^b^—*p* < 0.05 compared to the DM group.

Our results confirm the evidence of an increase of carbonyl-oxidative stress in the condition of diabetes (Figure 6 and Figure 7). In particular, analysis of H_2_DCFDA probe oxidation by comparative of the average values of fluorescence intensity showed that the level of ROS in leukocytes of diabetic rats is 1.5-fold higher than that in leukocytes of the control group of animals (Figure 6A). We observed a 1.4-fold reduction in the most abundant intracellular non-protein antioxidant in cells (i.e., GSH) compared to the control group (Figure 6B). In addition, there was a 23% increase in the level of LPO, which was estimated by the content of TBA-RS (Figure 6C).

The level of diabetes OMPs of neutral and basic nature also increased by 2.5-fold and 1.5-fold, respectively, compared to the control group (Figure 7A,B). Interestingly, leukocytes from diabetic animals showed 1.7-fold and 1.6-fold decrease in AOPPs and AGEs levels, respectively (Figure 7C,D).

Treating diabetic rats with LA led to a 1.3-fold increase in the level of GSH (Figure 6B). Simultaneously, the levels of intracellular ROS and TBA-RS decreased by about 17% both compared to non-treated animals (Figure 6A,C). The administration of the LA to diabetic animals in leukocytes led to lower levels of OMP_370_ and OMP_430_, reaching nearly 2- and 1.5-fold decreases, respectively, compared to the DM group (Figure 7A,B). The content of the AOPPs increased by 1.5-fold in LA-treated diabetic rats (Figure 7C), but the level of AGEs was not affected (Figure 7D).

## 4. Discussion

Our previous work showed the antidiabetic and antioxidant effects of extracts of red and yellow fruits of Cornelian cherries (*Cornus mas* L.) on rats with STZ-induced diabetes [6]. The antidiabetic properties of extracts from *Cornus mas* L. fruits were evidenced by a lowering of blood glucose, amount of glycated hemoglobin and improved glucose tolerance. Treatments with those extracts also increased the level of GSH, reduced the oxidative modifications of proteins and lipids, glycation, and oxidation protein formation or accumulation in plasma. *Cornus mas* L. active compounds are iridoids, anthocyanins, phenolic acids, and flavonols. One of the major iridoid glycosides in Cornelian cherries is LA. Due to the fact that the studied extracts contain iridoids in significant amounts (73.5% in red Cornelian cherry fruits, 88.2% in yellow Cornelian cherry fruits), the present study evaluated the antidiabetic and antioxidant properties of pure LA extract from *Cornus mas* L. fruits on diabetic rats.

We do not observe any hypoglycemic effects of LA at a dose of 20 mg/kg b.w. in rats with streptozotocin-induced DM (Figure 1). The reason could be due to a too low dose of LA. The administration dose and duration were selected based on previous studies [6]. We have supposed that this dose was important for conducting a comparative analysis of the effects of fruit extracts and their main iridoid glycoside. Consequently, we did not change the dose of LA in this experiment. In order to test whether LA has a hypoglycemic effect, we plan to investigate the dose effects of LA in STZ injected rats in the next experiments.

Fasting glucose levels, glycated hemoglobin, and OGTT data indicated that LA cannot improve the sensitivity or stimulate secretion of insulin in the STZ-induced diabetic rats (Figure 1). However, it was shown that oral administration of bitter root extract compounds such as LA, gentiopicrin and rindoside in db/db mice was associated with higher glucagon-like pepetide-1 (GLP-1) and lower blood glucose responses following glucose gavage [31,32]. In mice fed a high fat diet, oral administration of bitter gourd extract prior to an oral or intraperitoneal glucose load also resulted in higher GLP-1 and insulin levels and lower blood glucose responses [33]. Therefore, LA exhibits a hypoglycemic effect in the presence of functional Langergans islets in the pancreas. It is known, that GLP-1 induce expansion of insulin-secreting β-cell mass in pancreas, in addition to its ability to augment of glucose-stimulated insulin secretion. After conducting the morphological and histological studies of pancreatic islets, we found that there were no significant differences in the parameters of pancreatic islets such as area, volume, diameter and the number of β-cells of the pancreatic square (data not shown). The results indicate that LA did not have an influence on the islet architecture and did not restore of quantity and functional activity of β-cells of rats with STZ-induced diabetes.

According to our results, the increase of intracellular ROS level, TBA-RS and the content of carbonyl groups confirm the development of oxidative stress with subsequent activation of LPO and protein modification processes in leukocytes under diabetes (Figure 6A,B and Figure 7A,B). The administration of LA to diabetic animals led to a decrease in the lipoperoxidation products accumulation and lowers the content of OMPs in leukocytes (Figure 6B and Figure 7A,B). It was previously shown that the iridoids from lyophilized aqueous extract of *Ajuga iva* (Ai) were able to reduce oxidative stress and may prevent LPO in hypercholesterolemic rat [34]. The positive effects on lipid metabolism, via the modulation of PPARα and PPARγ expression, were also described for LA extracted from *Cornus mas* L. and iridoids extracted from *Cornus officinalis* L. [10,35,36,37,38].

Formation of OMP characterize by introduction of additional carbonyl groups into proteins. Carbonylation of proteins may be due to oxidation of side chains of some amino acid such as methionine, lysine and arginine or their interaction with ROS and non-oxidative reactions with oxidized lipids containing carbonyl groups [39]. Instead, we have also investigated the level of AOPPs in plasma and leukocytes, which are formed as a result of the reaction between chlorinated oxidants and proteins. We obtained quite interesting results that indicate a decrease of AOPPs in diabetic leukocytes (Figure 7C), although an increase in this indicator in the plasma was observed (Figure 4C). The major pathway leading to the formation of AOPPs is mainly dependent on myeloperoxidase (MPO) chlorination activities. Polymorphonuclear cell and monocytes contain the enzyme MPO which catalyzes the reaction of hydrogen peroxide with chloride ion to generate large amounts of hypochlorous acid, a powerful oxidizing and chlorinating agent [40]. Thus MPO is relevant to oxidative/chlorine stress that can alter lipids as well as proteins. It is known that the activity of MPO is inhibited in leukocytes under type 1 diabetes, resulting in diminished phagocytic activity of neutrophils and thus increasing susceptibility to infections. On the other hand, concentrations of MPO, which are stored intracellularly in primary azurophilic granules and liberated into the extracellular space after neutrophil activation and degranulation, increased in serum, which reflects the increased risk of diabetic complication [41].

Therefore, we suppose two possible models for our results. The first one assumes that a higher concentration of AOPPs in plasma (Figure 4C) is associated with increasing MPO enzyme levels in the plasma [42] and consequently associated with oxidative/chlorine stress, which leads to the accumulation of modified proteins in the condition of diabetes. Secondly, it is possible that a significantly lower concentration of AOPPs in leukocytes (Figure 7C) is associated with inhibited MPO activity in neutrophils [42]. It can also depend on the higher mean percentage of lymphocytes (80%) and lower mean percentage of neutrophils (16%) in diabetic rats compared to control rats [6]. Moreover, organic chloramines can be slowly hydrolyzed into aldehydes [40], thus increasing the pool of OMPs of neutral and basic nature in blood plasma and in leukocytes under DM (Figure 4A,B and Figure 7A,B).

Interestingly, a significant increase in AOPPs content in leukocytes towards the control value was observed in LA-treated animals (Figure 7C). At the same time, the concentration of plasma AOPPs remains at the same level as in diabetic rats (Figure 4C). In this regard, we can hypothesize that significantly higher concentration of AOPPs in plasma in comparison to leukocytes is the result of accumulation of modified proteins. The exact mechanism of LA action still requires further investigation.

We found that concentration of plasma AGEs remained at higher level in the LA-treated diabetic group (Figure 4D). AGEs-mediated cross-linking of plasma proteins is likely to be responsible for “metabolic memory” and therefore diabetic complications can continue long after achieving glycemic control [43]. This may suggest that AGEs accumulated in blood plasma because the administration of LA showed no hypoglycemic effect in diabetic rats (Figure 1).

Also, we investigated the levels of AGEs in leukocytes and observed a decrease in this indicator under diabetes (Figure 7D). These results can be related to decreasing glucose uptake from the bloodstream into the leukocytes. Glucose transport across the plasma membrane of leukocytes is the limiting step for its subsequent metabolism and depends on the function of specific glucose transporters (GLUT). Isoform GLUT1, which is expressed in lymphocytes, is non-insulin-dependent and transports reduced sugars such as glucose, mannose, galactose and glucosamine. However, GLUT3 and GLUT4 isoforms, also found on leukocytes, have higher affinity for glucose as compared to GLUT1. In leukocytes, GLUT3 and GLUT4 are localized in intracellular vesicles, which can translocate and fuse with the plasma membrane during cell activation by certain factors, including insulin in B-lymphocytes and monocytes. [44,45]. In this regard, we hypothesize that reducing translocation of GLUT3 and GLUT4 from intracellular vesicles to the plasma membrane in mononuclear leukocytes in conditions of hypoinsulinemia leads to a decrease in intracellular glucose level. In addition, the utilization of glucose is 2-fold lower in the diabetic than in healthy animals [46]. Moreover, energy production in blood leukocytes relies on catabolism of fatty and amino acids through tricarboxylic acid cycle while glycolysis is of minor importance [47]. For instance, glucose utilization by plasma cells amounted to 0.5 μmol/h × 10^8^ cells and was mainly via the Embden-Meyerhof pathway and only 6% or less traversed the pentose phosphate pathway [48]. This suggests that in conditions of lower glucose concentration in leukocytes is probably less intensified the formation of reactive carbonyl species as a group of highly reactive metabolites and results in decreased formation of AGEs. The administration of LA to animals with DM led to an increase in the content of AGEs to values of the control group (Figure 7D).

The decreased concentration of oxidative stress biomarkers in leukocytes confirms the potential antioxidant efficiency of LA (Figure 6 and Figure 7). Therefore, LA inhibits the generation of ROS and LPO. The free radical scavenging activity of LA was also demonstrated by Abirami et al. [49].

Wide ranges of antioxidant mechanisms rely on GSH as a co-substrate during the detoxification process with increased amounts of ROS [47]. Reduction of GSH concentration in leukocytes (Figure 6A) confirms the disturbance of redox homeostasis and antioxidative capacity at the cellular level in rats with STZ-induced diabetes. In conditions of hyperglycemia, the activation of the sorbitol pathway of glucose metabolism and NADPH-dependent oxidase leads to a decrease in GSH concentration. Increased consumption of GSH consecutively impairs the glyoxalase system, enzymes of which metabolize methylglyoxal (represents a reactive carbonyl species) into lactate by using NADPH and GSH [47]. Under the administration of LA to animals with diabetes, increasing the concentration of GSH (Figure 6A) may be involved in lipid peroxides neutralization and maintenance of SH-groups of proteins in the restored state in leukocytes.

Impaired glucose metabolism due to diabetes and oversaturation of the electron transport chain of cells leads to the overproduction of superoxide anion and its derivatives. It is known that oxidative stress plays a significant role in the pathogenesis of diabetes and causes protein modification and subsequent complications. Antioxidant enzymes have shown an important role in maintaining physiological levels of superoxide anion and hydrogen peroxide by dismutation of oxygen radicals and eliminating organic peroxides and hydroperoxides [50]. Our experiments correlate with previous data and studies [51] and show a decrease in the activity of antioxidant enzymes under the condition of STZ-induced diabetes in rats (Figure 5). The activity of enzymes may be affected by the processes of direct or indirect oxidation in conditions of excess of ROS, and ROS-mediated modification of proteins under diabetes, such as nitration [51]. These processes have a negative effect on the cell components, protein molecules, including enzymes, and leads to disruption of their structure and an alteration of certain properties.

Compounds that alleviate oxidative stress may be beneficial against complications related to diabetes. We found that LA improves the antioxidant status of leukocytes via increasing the activities of CAT, GPx, and GR (Figure 5). Administration of LA to animals with diabetes led to decreasing the levels of oxidative markers such as TBA-reactive substances (Figure 6B) and oxidative modifications of proteins of neutral and basic nature (Figure 7A,B). However, decreased in SOD activity under diabetes was not showed by LA administration (Figure 5A). Such results may be explained by the fact that SOD is an enzyme of the first line of defense, which plays a major role in the elimination of ROS. The oxidant-antioxidant imbalance at the direction of ROS and NO overproduction [51] leads to intensive formation of peroxynitrite in leukocytes of rats with DM. Superoxide-anion reacts with nitric oxide at a 3- to 5-fold faster than the dismutation process of O_2_^•−^, catalyzed by SOD. Peroxynitrite may inactivate SOD via nitration, dityrosine cross-linking formation and disruption of the Cu, Zn-complex with further increasing O_2_^•−^ production [52]. So, this enzyme is sensitive to oxidative-nitrative stress and may undergo ROS-dependent inactivation, thus its activity is decreased.

LA showed not only antioxidant properties but also possess anti-inflammatory activity by activation of AMP-activated protein kinase (AMPK) [53]. AMPK is a regulator of metabolic pathways and inflammatory activity in leukocytes. Studies of AMPK in leukocytes have contributed to the emergent paradigm that reduced glycolysis and enhanced oxidative metabolism are associated with suppressed inflammation, whereas increased glycolysis is associated with inflammatory activity [54]. Thus, an increase in AMPK activity is accompanied by decreased cellular glucose consumption [55]. Activated AMPK suppresses inflammatory responses and repolarize leukocytes by inducing anti-inflammatory gene expression programs through the Akt/mTORC1 and JAK/STAT signaling pathways [54]. The antioxidant effect of LA also can be dependent on the activation of AMPK and the resulting induction of PPARγ coactivator-1a (PGC-1a) and Mn-SOD [56].

## 5. Conclusions

We provide evidence that LA, an iridoid glycoside extracted from *Cornus mas* L. fruits, exhibits antioxidant properties in relation to STZ-induced DM. This conclusion is evidenced by a decrease in some biomarkers of oxidative stress, such as level of protein carbonyl groups in plasma, and TBA-reactive substances and OMPs of neutral and basic nature in leukocytes. The administration of LA to diabetic animals also led to an increase in the activity of antioxidant enzymes like CAT, GPx, GR and, as a result, increasing GSH levels in blood leukocytes. However, iridoid did not show a hypoglycemic effect on the streptozotocin model of diabetes in rats. Such results may indicate LA as a potential antioxidant agent and need further investigation. Considering LA is a native component in red and yellow fruits of *Cornus mas* L. common in the territories of southern and central Europe and southwest Asia, it could be a food supplement and a safe plant therapeutic agent to alleviate oxidative stress associated with DM.

## Figures and Tables

**Table 1:** Identification and the content (mg/100 g dw) of the compounds of the purified LA extract from cornelian cherry fruits by LC-MS and HPLC-PDA.

Compound	UV λ_max_ (nm)	[M−H]^−^ (*m/z*)	Other Ions (*m/z*)	Content (mg/100 g dw)
**Iridoids**				
Loganic acid 1	246	375	213	183.1 ± 27.4
Loganic acid 2 ^a^	246	375	213	70,228.5 ± 548.6
Loganic acid 3	246	375	213	3581.7 ± 73.1
***Total***				**73,993.3**
**Phenolic acids**				
Caftaric acid 1	327	179/311	149/135	721.7 ± 10.1
Caftaric acid 2 ^a^	328	179/311	149/135	1110.2 ± 20.0
***Total***				**1831.9**

^a^—Compounds identified by comparing retention time, absorption and mass spectra with that of commercial standards.

**Table 2:** Effect of loganic acid on number of leukocytes and differential leukocyte count in diabetic rats.

Parameters	Groups		
	Control	DM	DM + LA
Leukocytes, 10^3^/µL	10.1 ± 0.8	11.2 ± 0.7	12.0 ± 0.8
Banded neutrophilis, %	1.4 ± 0.2	1.3 ± 0.4	1.5 ± 0.3
Segmented neutrophilis, %	21.7 ± 1.3	16.3 ± 1.6 ^a^	19.2 ± 1.8
Eosinophils, %	1.2 ± 0.4	1.0 ± 0.3	1.3 ± 0.3
Lymphocytes, %	74.4 ± 1.5	80.4 ± 1.3 ^a^	77.5 ± 2.6
Monocytes, %	0.5 ± 0.2	0.4 ± 0.3	0.5 ± 0.1

^a^*p* < 0.05 compared to the control group.
